# Supplementary material for: The interactome of intact mitochondria by cross-linking mass spectrometry provides evidence for coexisting respiratory supercomplexes
Source: Mol Cell Proteomics. 2017 Dec 8;17(2):216–32. doi: 10.1074/mcp.RA117.000470 (PMC5795388; doi:10.1074/mcp.RA117.000470)
Supplement: Supplemental Data [file supp_RA117.000470_133922_0_supp_23978_fzffwf.zip › spectra_annotation/mito_spectra_annotation/377-1-11-1-11-1.pdf]

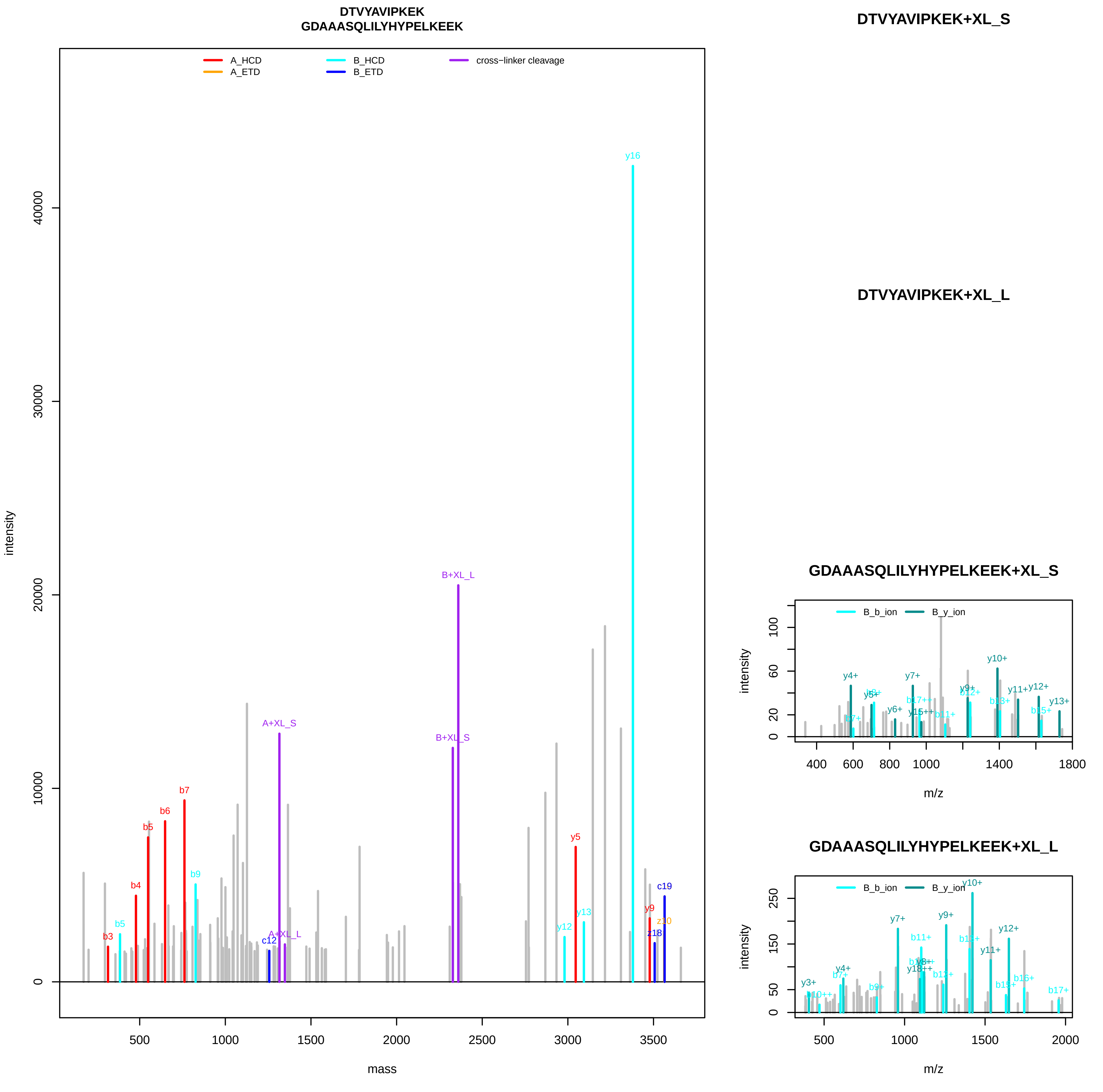
DTVYAVIPKEK
GDAAASQLILYHYPELKEEK
A_HCD
A_ETD
B_HCD
B_ETD
cross−linker cleavage
intensity
mass
0
10000
20000
30000
40000
500
1000
1500
2000
2500
3000
3500
y16
B+XL_L
B+XL_S
A+XL_S
A+XL_L
b3
b4
b5
b6
b7
b9
c12
y5
y9
y12
y13
z18
c19
z10
DTVYAVIPKEK+XL_S
DTVYAVIPKEK+XL_L
GDAAASQLILYHYPELKEEK+XL_S
B_b_ion
B_y_ion
intensity
m/z
0
20
60
100
400
600
800
1000
1400
1800
y4+
y5+
y6+
y7+
y9+
y10+
y11+
y12+
y13+
b7+
b12+
b13+
b15+
GDAAASQLILYHYPELKEEK+XL_L
B_b_ion
B_y_ion
intensity
m/z
0
50
150
250
500
1000
1500
2000
y3+
y4+
b7+
y7+
b9+
b11+
y9+
y10+
y11+
y12+
b16+
b17+
